# Oxytocin improves synchronisation in leader-follower interaction

**DOI:** 10.1038/srep38416

**Published:** 2016-12-08

**Authors:** L. Gebauer, M. A. G. Witek, N. C. Hansen, J. Thomas, I. Konvalinka, P. Vuust

**Affiliations:** 1 Center for Music in the Brain, Dept. of Clinical Medicine, Aarhus University & The Royal Academy of Music Aarhus/Aalborg, Denmark; 2 Interacting Minds Centre, Aarhus University, Denmark; 3 Department of Psychology and Behavioural Sciences, Aarhus University, Denmark; 4 School of Communication and Culture, Aarhus University, Denmark; 5 Faculty of Psychology and Neuroscience, Maastricht University, Netherlands; 6 Section for Cognitive Systems, Department of Applied Mathematics and Computer Science, Technical University of Denmark, Denmark

## Abstract

The neuropeptide oxytocin has been shown to affect social interaction. Meanwhile, the underlying mechanism remains highly debated. Using an interpersonal finger-tapping paradigm, we investigated whether oxytocin affects the ability to synchronise with and adapt to the behaviour of others. Dyads received either oxytocin or a non-active placebo, intranasally. We show that in conditions where one dyad-member was tapping to another unresponsive dyad-member – i.e. one was following another who was leading/self-pacing – dyads given oxytocin were more synchronised than dyads given placebo. However, there was no effect when following a regular metronome or when both tappers were mutually adapting to each other. Furthermore, relative to their self-paced tapping partners, oxytocin followers were less variable than placebo followers. Our data suggests that oxytocin improves synchronisation to an unresponsive partner’s behaviour through a reduction in tapping-variability. Hence, oxytocin may facilitate social interaction by enhancing sensorimotor predictions supporting interpersonal synchronisation. The study thus provides novel perspectives on how neurobiological processes relate to socio-psychological behaviour and contributes to the growing evidence that synchronisation and prediction are central to social cognition.

As interacting agents, humans have an extraordinary ability to adjust their behaviour and beliefs to others. In recent years, the neuropeptide oxytocin (OT) has received extensive interest in social neuroscience due to its effects on social bonding^1^, trust^2^, cooperation^3^,^4^, group conformity^5^, parenting^6^, empathy^7^, and emotion recognition^8^. However, the psychological mechanism through which OT exerts these effects is unclear^9^. OT is often referred to as a ‘social hormone’ and has been suggested to *directly* improve higher order social cognition. Alternatively, OT has been suggested to enhance social behaviours *via* a reduction in stress and/or anxiety^10^, or *via* increasing the salience of social cues^11^.

Here we put forth the alternative hypothesis that OT affects social dynamics by facilitating interpersonal synchronisation via prediction and/or adaptation. Our hypothesis is supported by previous studies showing that OT increases mu wave suppression when perceiving biological motion, which may be indicative of increased motor simulation, i.e. the neural modelling of others’ behaviour, enabling inferences about their future actions^12^. OT has also been found to enhance behavioural and affective synchrony in parent-infant interaction^6^, motor simulation^13^, and is released during choral singing^14^,^15^. Furthermore, Arueti *et al*.^16^ found that OT improved interpersonal coordination in an ‘etch a sketch’ task, but only for a small subset of their participants. By testing the effect of OT on interpersonal *rhythmic* synchronisation, we provide novel insights into the role of OT in enhancing synchronisation in social dynamics.

The ability to predict the behaviour, thoughts, and feelings of others is essential for successful social interaction^17^,^18^. The more similar other people are to oneself, the easier it is to simulate their state, leading to more successful predictions^18^,^19^. Consistent with this, people tend to synchronise spontaneously in social contexts, for example by walking in time^20^, or synchronising handclapping^21^. Interpersonal synchronisation is defined as alignment of periodic behaviour with another person^22^. This has been shown to promote pro-social behaviour^23^, such as helpfulness^24^, liking^25^,^26^, cooperation^27^ and trust^28^,^29^, even when the outcome of that behaviour is anti-social towards a third person or group^30^.

Online prediction^31^,^32^ and adaptive error correction^33^ are fundamental to synchronisation, since accurate alignment of movements requires the projection of future events based on past events and continuous motor adjustments. For example, performers in a musical ensemble must be able to anticipate each other’s temporal phrasing in order to achieve an acceptable level of synchronisation^32^. It has been shown that making oneself more predictable, by reducing temporal variability, is an effective strategy for improving interpersonal coordination^34^. Finger-tapping studies provide a well-established way of investigating behavioural synchronisation with an external referent, such as a metronome, as well as with other people^35^. In social contexts, prediction supports coordination and synchronisation of ones own movements with those of another person. It has been suggested that motor simulation enables prediction of another person’s actions based on internal models in one’s own motor system^13^,^19^,^36^,^37^.

While social and non-social finger-tapping may implicate the same prediction mechanisms, prediction of another person’s behaviour may be more challenging than a regular computerized rhythm. Using an interactive finger-tapping task, Konvalinka, *et al*.^31^ showed that people were mutually adaptive on a tap-by-tap basis when synchronising interpersonally. When tapping to a variable but responsive partner (bidirectional tapping), participants were equally well synchronised as when tapping to a non-variable and nonresponsive computer metronome. However, synchronisation was significantly impaired with a partner that was both variable and nonresponsive (unidirectional tapping), i.e. when there was a predetermined leader-follower relationship. In other words, coordination is facilitated by prediction of another person’s timing behaviour, and reciprocity (adaptability) of the interactive partner.

Despite the widespread recognition of the influence of both OT and interpersonal synchronisation on social behaviours, no study has looked at the relation between OT and interpersonal synchronisation. This was the aim of our study, adopting an interpersonal tapping paradigm as a model of minimal social interaction^31^. Dyads were administered either OT or a non-active placebo intranasally. Both dyad members always received the same solution and were seated in two separate closed-off rooms with headphones through which auditory feedback was provided. They were instructed to tap along to a beat in three conditions varying in degree of social interaction; computer, bidirectional and unidirectional condition (see Fig. 1). Participants received the following instructions: 1) ‘The metronome will not stop. Continue synchronizing your tapping to the beat of the **metronome’** (computer condition: both hear completely regular computer-generated beats). 2) ‘After the metronome stops you will hear the **other participant** tapping. Try to keep the beat together, and synchronize to your partner’s tapping as much as possible’ (bidirectional condition: both participants hear beats generated by the other participant, but not their own tapping. Unidirectional following: one participant hears the tapping of the other, who is self-pacing (i.e. he is in condition 3)). 3) ‘After the metronome stops, you will hear **yourself** tapping. Try to stick to the beat you heard from the metronome’ (unidirectional leading: the participant hears his own tapping, and acts as leader for the other participant who is in condition 2).

From the recorded MIDI data, the following measures were extracted: Synchronisation index (SI); Tapping variability, calculated as standard deviation of inter-tap-intervals (ITIs); Tapping speed, measured as mean ITI; Amount and magnitude of positive and negative asynchrony during unidirectional tapping, indicating whether tappers reacted to or anticipated (tapped prematurely in relation to) the other’s tapping; Adaptability, represented by windowed cross-correlations between ITIs of paired members. This paradigm allows for an investigation of OT’s effect on interpersonal synchronisation on a millisecond time scale. Questionnaires recorded mood, liking of tapping partner and personality measures.

We hypothesized that there would be an interaction between group and condition, i.e. that dyads given OT would show increased synchronisation compared to dyads given placebo, but only in conditions that involved interacting with another person. We were also interested in whether OT would specifically affect prediction (as indexed by variability of ITIs) and/or adaptation (+1/−1 cross-correlation coefficients between dyad members); and similarly whether OT affected interpersonal synchronisation through premature/anticipatory (negative asynchronies) or reactive (positive asynchronies) mechanisms. By asking about a tapping-partner’s liking and mood, we also addressed the extent to which OT affected higher-order social cognition or improved social interaction via anxiety and stress reduction.

Increasingly, it has also become clear that personality may interact with the pro-social effects of OT^11^. In one study^16^, OT only improved coordination for competitive participants, and in another^38^, OT was only beneficial to less emotionally/socially competent people. It thus seems that OT might act as a compensation for the lack of socially constructive skills. Therefore, we expected that OT’s effect on synchronisation would interact with personality, i.e. that less empathic/socially competent persons would show a greater benefit of OT. These personality factors were measured using the empathizing and systemizing quotient (EQ^39^ and SQ^40^), as well as the Toronto Alexithymia Scale (TAS^41^).

## Results

### Synchronisation indices

Synchronisation indices (SIs) were used to analyse how well pairs synchronised during the regular computer-generated beat, during bidirectional tapping (where both pair-members could hear the other) and during unidirectional tapping (where only one of the pair-members could hear the other). The SI is a unitless number, which ranges from 0 to 1, representing the absence of synchronisation and perfect synchrony, respectively. Since the SI is a measure of the relative phase between two signals, here the tapping of two participants (or one participant and the computer), it is not possible to calculate individual measures for the bidirectional and unidirectional condition, but each dyad has a shared value describing how well they synchronise their tapping. This also meant we could not take the covariates (participants’ individual SQ and TAS scores) into account for this analysis. Using a linear mixed effects model (LMM), with dyads entered as a random factor, and group, condition, group × condition as fixed-factors, we found significant main effects of condition and group, and a significant interaction between group and condition (Table 1). As depicted in Fig. 2, Bonferroni-corrected post-hoc tests showed that the OT group synchronised significantly better than the placebo group in the unidirectional condition (*p* = 0.015), with no significant effects of OT in the bidirectional (*p* = 0.790) and computer conditions (*p* > 0.999). Participants in both groups were most synchronised in the computer condition, followed by the bidirectional and unidirectional conditions, respectively (all *p* < 0.001).

### Tapping variability

Standard deviations (SD) of inter-tap-intervals (ITI) were used to measure the temporal variability of tapping, using individual responses as the analysis units. We performed an LMM to investigate the effects during the computer and bidirectional conditions, with individual subjects as random factors, and group, condition, group × condition, group × SQ and group × TAS as fixed factors. Here we found a significant main effect of condition *F*(1, 91) = 23.20, *p* < 0.001, but no effect of group *F*(1, 90) = 2.37, *p* = 0.127. There was also a significant main effect of SQ *F*(1, 91) = 5.25, *p* = 0.024, with a negative coefficient (−0.00022). Bonferroni-corrected post-hoc tests showed that tappers were significantly more variable in the bidirectional (Mean = 0.040, SE = 0.002) than the computer condition (Mean = 0.025, SE = 0.002, *p* < 0.001). In the unidirectional condition, we split the groups into ‘leading’ (tap to self) and ‘following’ (tap to unresponsive other) and performed an LMM. We used ‘interacter-role’ (leading vs. following) as the within-subjects variable, since both dyad members were given the opportunity to act as both leader and follower. Individual subjects were the random factor, and the following were specified as fixed factors: group, role, group × role, group × SQ and group × TAS. We found a significant main effect of role (leading vs. following) *F*(1, 91) = 16.85, *p* < 0.001, and a significant interaction between role and group *F*(1, 91) = 5.61, *p* = 0.020. There was also a marginally significant main effect of SQ *F*(1, 90) = 3.38, *p* = 0.070, with a negative coefficient (−0.00021). As depicted in Fig. 3a, Bonferroni-corrected post-hoc tests showed that there was no significant differences between groups in leading variability *p* > 0.999, but a marginally significant difference in following variability *p* = 0.058. Furthermore, variability was significantly greater for followers than leaders in the placebo group *p* < 0.001, but in the OT group, the difference was not significant *p* = 0.660. This suggests that, when controlling for self-paced leading variability, followers given OT were less variable than followers given placebo. Figure 3b,c exemplify tapping variability in single dyads of the two groups, respectively.

### Tapping speed

For tapping speed (mean ITI), we performed an LMM with individual subjects as the random factor and the following as fixed factors: group, condition, group × condition, group × SQ and group × TAS. Since we found no effects of SQ or TAS, we reran the model without these covariates. The reduced model was not significantly different from the full model. We found a significant main effect of condition *F*(2, 192) = 70.48, *p* < 0.001 and a significant interaction between group and condition *F*(2, 192) = 3.93, *p* = 0.021. Bonferroni-corrected post-hoc tests demonstrated significant differences between the computer condition and bidirectional tapping, and between unidirectional and bidirectional tapping for both groups, all *p* < 0.001, with the longest mean ITIs in the computer condition, followed by the unidirectional condition, and the fastest ITIs in the bidirectional condition (See Fig. 4a). The shorter ITIs in the bidirectional condition were most pronounced in the OT group, which showed significantly lower mean ITIs compared to the placebo group *p* = 0.011. There were no significant differences between OT and placebo groups for neither the unidirectional condition *p* > 0.999, nor the computer condition *p* > 0.999. Figure 4b,c show tapping speed for a single dyad in the bidirectional condition, in the oxytocin and placebo groups, respectively.

### Mutual adaptation

We found no effect of OT on mutual adaptation patterns, measured with windowed cross-correlations, in any of the conditions. Ignoring group, the results for lag −1, 0 and +1 were according to those reported in Konvalinka *et al*.^31^, with the unidirectional condition showing only a positive lag −1 OR lag +1 cross-correlation (depending on which member was leading), and the bidirectional condition showing both positive lag −1 and lag +1 cross-correlations, and a negative lag 0 correlation, indicating mutual adaptation.

### Asynchronies

To analyse if the member acting as follower in the unidirectional condition demonstrated more premature/anticipatory tapping errors (i.e. tapped before the leader) or more reactive tapping errors (i.e. tapped after the leader), we calculated negative and positive asynchronies and performed separate LMMs, as well as correlations with SIs. As with SIs, asynchronies depend on the relation between the tapping of two dyad members, one who is self-paced (leading) and one who is trying to adapt (following). Thus what represents a negative asynchrony for one (i.e. the one following) corresponds to an equal positive asynchrony for the other tapper (i.e. the one leading). Since only the participant following can adapt his tapping to the other participant, we report the asynchronies based on the participant who was following. There were no effects of SQ or TAS on either positive or negative asynchronies, and the LMMs with SQ and TAS as covariates were not significantly different from the LMMs without covariates. We therefore report results from the LMMs without covariates. For negative asynchronies, there was no group difference. For positive asynchronies, there was a marginally significant effect of group *F*(1, 96) = 3.19, p = 0.077, suggesting a trend that the magnitude of positive asynchronies was smaller for the OT group (Fig. 5). We also performed LMMs (again, the covariates were not significant, hence we report the reduced model) on the number of positive vs. negative asynchronies for the two groups. There was no group difference, but a significant effect of asynchrony category *F*(96, 1) = 200.42, *p* < 0.001, showing that the asynchronies were more often negative (Mean = 202.57, SE = 6.09) than positive (Mean = 80.80, SE = 6.09). For both groups there were significant correlations between synchronisation indices and positive and negative asynchronies, respectively (SI and positive asynchronies: OT r = −0.437, p = 0.002; placebo r = −0.773, p < 0.001. SI and negative asynchronies: OT r = 0.506, p < 0.001; placebo r = 0.515, p < 0.001). Between the two groups, the correlations were significantly different for the positive asynchronies (SI and positive asynchronies z = 2.65, p = 0.008), suggesting that the correlation between synchronisation and positive asynchronies was stronger for the placebo group than the OT group. The correlations were not significantly different for the negative asynchronies. There were also strong negative correlations between variability (SD) of absolute asynchronies and SIs for both the OT (*r* = −0.92, *p* < 0.001) and the placebo group (*r* = −0.96, *p* < 0.001), indicating that the more synchronised the tappers, the more stable their interaction overall.

### Mood, liking and personality

No effect of OT was found on mood or liking of tapping partner. SQ neither correlated with EQ (*r* = 0.156, *p* = 0.139) nor with TAS (*r* = *−*0.021, *p* = 0.843). EQ and TAS correlated (*r* = −0.535, *p* < 0.001), and we therefore excluded EQ from further analysis and just focused on TAS and SQ, since this gave us the highest possible number of complete subject data (N = 93). Finally, participants were not able to guess whether they had been given oxytocin or placebo.

## Discussion

Using an interactive finger-tapping paradigm, we show for the first time that oxytocin (OT) improves synchronisation when interacting in a predetermined leader-follower relationship (unidirectional condition). This improved synchronisation could be explained by the reduced variability in following behaviour in the OT group. In this condition, there was also a borderline significant effect on positive asynchronies where placebo tappers produced greater positive asynchronies than OT tappers, indicating a reduction in reactive tapping errors in the OT group. However, there was no effect of OT on adaptation patterns, as measured using windowed cross-correlations.

The effect of OT on synchronisation, temporal variability and asynchronies suggests that OT improves the ability of a follower to predict the tapping of an unresponsive leader. In order to better align their own actions with their partner’s, followers must more accurately predict the actions produced by their partners^35^,^42^. When the partner is unresponsive, OT may help with such predictions. The improved predictions are reflected in the reduced tapping variability of following behaviour and the overall increased synchronisation between leader and follower. Furthermore, a reduction in the magnitude of positive ‘follower’ asynchronies for the OT group, albeit marginally significant, indicated a trend that the taps of the OT group were closer to their tapping targets (the leaders’ taps), suggesting that they better predicted the correct timing of the leaders, allowing them to synchronise better. The significantly stronger negative correlation between synchronisation and positive asynchronies for the placebo group (compared to the OT group) further suggested that without the added OT, synchronisation is more strongly driven by reactive tapping. These observations further strengthen our conclusion that OT improves prediction in low-level social interaction. Unsurprisingly, synchronisation correlated with both positive and negative asynchronies for both groups, but the correlation was stronger for placebo tappers with regard to positive asynchronies. We found no significant differences between the two groups in the number of negative vs. positive asynchronies, but overall tappers made more negative than positive asynchronies. This finding reflects the general *mean negative asynchrony* found between tappers and their tapping target in a wide range of tapping studies^35^.

In accordance with Konvalinka *et al*.^31^, bidirectional tapping was more synchronised than unidirectional tapping, because in the former, both tappers were able to hear and adapt to each other, while in the latter, only one tapper was adjusting his behaviour. The unidirectional interaction thus places higher demands on on the individual’s ability to predict to predict and adapt to the behavioural pattern of their irregular partner. This may explain why we do not see a significant effect of OT in the bidirectional condition. In situations where both agents are responding to each other, synchronisation is already facilitated via the mutual adaptation and it seems OT can do little to boost this already highly synchronised interaction. In other words, we might have a ceiling effect in our bidirectional condition. Notably, the synchronisation was higher and tapping variability lower in the OT group for the bidirectional condition also, though the difference was not significant. When there is only one person responding to another, as in the unidirectional interaction, there is more room for improvement, and OT may facilitate sensorimotor prediction of an unresponsive partner’s actions.

The decreased difficulty may also explain why we found no effect of OT when tapping to a computer, which was unresponsive, completely regular and thus maximally predictable. Indeed, it may be that tapping performance in the computer condition reached a ceiling effect, so no potential benefit of OT could be observed during this condition. It is therefore possible that OT improves synchronisation only when task difficulty is high, independently of whether the tapping-partner is human or non-human. Meanwhile, people tend to attribute signals that are variable and adaptive to other humans^29^,^43^, irrespectively of whether they are generated by a computer or by another person, making the arbitration between social and non-social signals less clear. Alternatively, OT may not improve synchronisation with distinctively non-human referents, such as a computer, because it affects social tasks exclusively^3^. This interpretation would be in line with previous studies showing that OT affects interpersonal imitation, but does not influence congruency on non-social tasks^13^. In order to determine whether OT interacts with task difficulty or level of social interaction, a study that controls for both of these factors in a tapping task is needed. An interesting framework for untangling the effects of task difficulty and social dynamics is Bayesian computational modelling, which has successfully been used in other social tasks^44^.

In the bidirectional condition, OT dyads tapped faster than placebo dyads, and further away from the given tempo. Note that the task was to synchronise *as well as* keep the tempo. Thus, OT pairs seem to have weighted the social task of synchronising with each other higher than keeping the given tempo. Furthermore, the OT group was not significantly less variable than the placebo group in the bidirectional condition. This is probably because following entrainment to the tempo of the initial metronome, tappers *gradually increased* the tapping tempo together, thus affecting their average tapping variability. This speeding up could arise from predictive processes related to sensory attenuation of ones self-generated tapping, which might be accentuated by OT^12^,^45^. Thus in the bidirectional condition, OT tappers are attenuating the feedback from their own tapping, thus ignoring how their own speeding up affects the tapping timing of their partners, causing a cumulative effect on overall tapping tempo. Sensory attenuation may also explain the improved synchronization for the OT group in the unidirectional condition. Since the leader’s tapping is independent of the follower’s (i.e. the leader cannot hear the follower and is unresponsive), the follower can more accurately predict the leader’s tapping when OT attenuates the perception of his own tapping.

OT did not change the pattern of adaptability, but seemed only to affect prediction as measured via synchronisation, variability and speed. We also did not find any effect of personality on the added effect of OT, suggesting that at a low level, oxytocin benefits synchronisation regardless of social and emotional competence. We did see an overall effect of the personality trait ‘systemizing’ on variability in the computer and bidirectional condition (and also a marginally significant effect in the unidirectional condition), where ‘high systemizers’ showed decreased tapping variability. ‘High systemizers’ might be able to structure their tapping better than ‘low systemizers’ causing lower variability across all conditions. This effect of personality might be related to altered predictive processing in people high in systemizing^46^,^47^.

Despite OT being repeatedly shown to affect subjective reports of pro-social attitudes, we did not find a significant effect of OT on liking of tapping partner. Thus, our data indicate that rather than having a direct effect on specialized higher-order social behaviour, OT primarily affects low-level behavioural interaction outside the participants’ conscious awareness, i.e. their ability to follow and predict the taps of an unresponsive partner. We suggest that this effect may mediate pro-social attitudes^2^,^3^,^7^. It is possible that a stronger connection between dyad members would have yielded greater effects on liking. In particular, only being able to interact through auditory coupling of tapping may have prevented fully-fledged subjective liking. In other words, our results do not suggest that OT has no social relevance. Rather, we contribute to the debate by showing that even in the absence of subjective experience of affiliation, OT affects behavioural synchronisation in leader-follower interaction.

Another mechanism that has been suggested to mediate pro-social effects of OT is anxiety and stress reduction^9^,^10^. While we did not directly measure anxiety or stress, we did ask for ratings of mood before and after OT/placebo administration and the tapping task. We did not find a significant change in mood, neither in the OT nor the placebo group. While it is possible that changes in mood or anxiety levels might be too subtle to be measured using questionnaires, our findings do not indicate that anxiety reduction is the primary mediator of the pro-social effects of OT. This supports our suggestion that the primary effect of OT is on sensorimotor prediction, temporal variability and synchronisation itself.

In conclusion, we show that in an interactive tapping task, oxytocin improves synchronisation and reduces tapping variability when one tapper is leading and the other following. These findings suggest that oxytocin improves sensorimotor prediction during unidirectional coupling. We thus contribute to the on-going debate regarding the mechanism behind oxytocin’s pro-social effects, and highlight the importance of prediction and synchronisation in social interaction more broadly.

## Methods

### Participants

One hundred participants were recruited through databases at Aarhus University, Denmark. The study was approved by the Central Denmark Region Committees on Health Research Ethics, and informed consent was obtained from all participants, who received a remuneration of 200 DKK. Methods were carried out in accordance with approved guidelines by the Helsinki Declaration. Only males were included since hormonal fluctuations in females affect endogenous OT levels^48^. All participants were non-musicians, defined as having no more than four years of formal or informal (self-taught) music training, and not having practiced music actively in the last four years. Participants were randomly assigned to either the OT or placebo group. One dyad from the placebo group had to be excluded since the two participants knew each other in advance, giving a total of 50 subjects (25 dyads) in the OT group and 48 subjects (24 dyads) in the placebo group (Table 2).

### Materials and Apparatus

Before arriving at the lab, participants completed three personality questionnaires online: The Empathizing Quotient (EQ)^49^, The Systemizing Quotient (SQ)^50^, and the Toronto Alexithymia Scale (TAS)^41^,^51^. EQ and SQ measure autistic traits, particularly related to mentalizing, attention to detail, and strong interests in patterns and systems. The TAS-20 measures the ability to identify and describe feelings, and externally oriented thinking. At the lab, current mood ratings were provided on a pre-test questionnaire, using a 7-point Likert scale. Participants then performed the rhythm sub-test of the Musical Ear Test^52^, assessing baseline rhythmic skill via a discrimination task. A post-test questionnaire asked participants to provide further demographics and ratings of current mood, tapping partner and task liking, and guess whether they had been given OT or placebo.

Upon careful instructions, either the synthetic OT analogue Syntocinon^®^ or a non-active placebo nasal spray (isotonic saline 0.9%) was self-administered intranasally to both dyad members. Each participant received a total dose of 24 IU for the OT dyads^10^,^53^. Following guidelines by Guastella *et al*.^53^, they took six bursts – three in each nostril in an alternating fashion – leaving approximately 10 sec between each burst.

Tapping data were recorded and stimuli presented following the procedure of Konvalinka *et al*.^31^. Tapping was recorded on Yamaha keyboards, and a mixer was used to control auditory feedback; specifically, participants heard via headphones either 1) the computer-generated metronome, 2) feedback from their own key presses, or 3) feedback from their partner’s key presses. Before each trial, participants always heard an 8-beat long metronome indicating the intended tapping tempo, 120 bpm. The elaborate set-up ensured an auditory delay time between pressing the key and hearing the sound of no more than 6 ms.

### Task and Procedure

The first part of the study took place in a room with both dyad members present. They were introduced and told that they would be ‘tapping partners’ during the main experiment. They then stayed in the same room, but performed tasks and completed questionnaires individually. Pre-test questionnaire and the MET test (~8 mins) were completed. Participants were given instructions to self-administer the nasal spray and then wait for 30 min while watching a documentary about glaciers (i.e. with no social relevance), separately on two laptops with headphones, to allow OT to be taken up by the CNS^9^.

**Figure 1:**
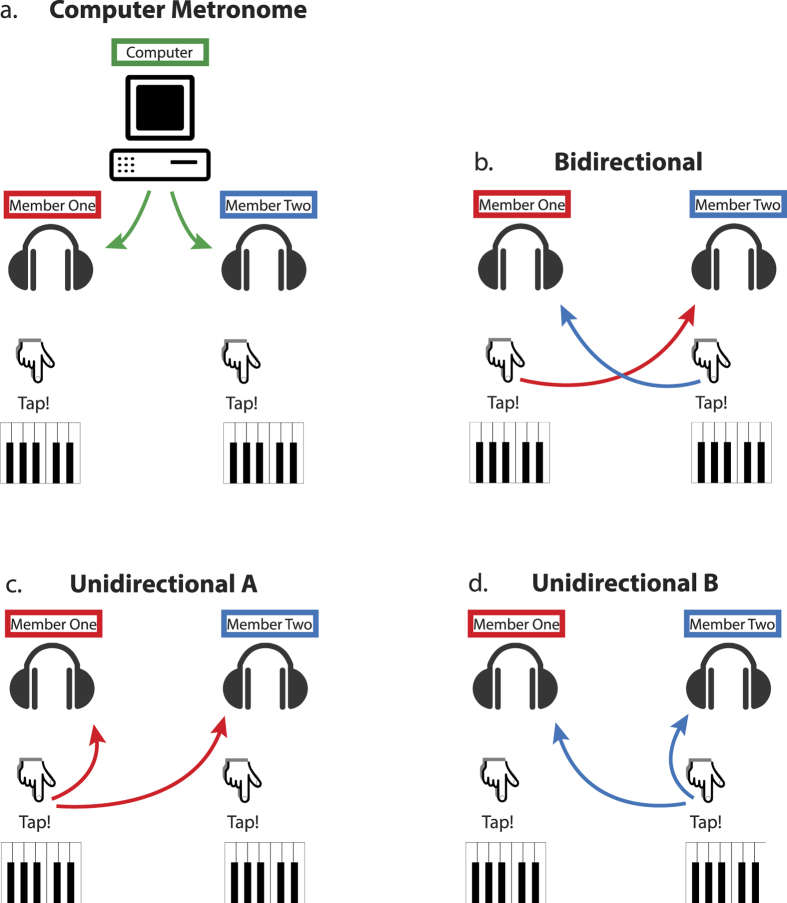
Tapping conditions with different degrees of social interaction: (**a**) computer condition/non-social condition where both dyad members hear and tap to regular computer generated beats with no variability; (**b**) bidirectional coupling/tapping to responsive other where both participants hear and tap to beats generated by the other participant, but not their own tapping; (**c,d**) unidirectional coupling/tapping to non-responsive other where both participants hear and tap to only the tapping of either member one (**c**) or member two (**d**), causing a leader-follower relationship. Auditory feedback is indicated with arrows. Green indicates the computer, red indicates member one, and blue member two.

**Figure 2:**
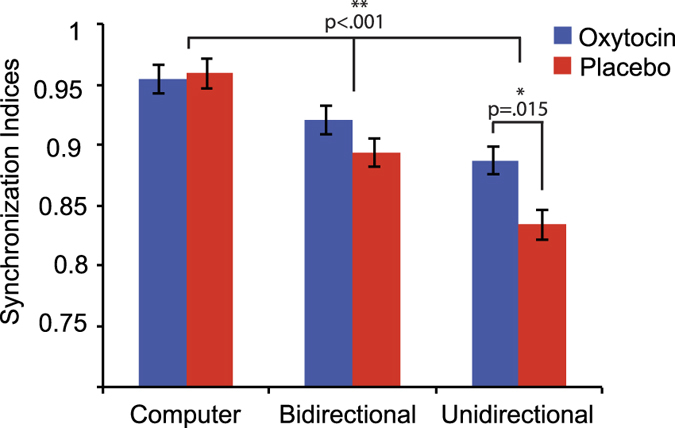
Effect of oxytocin vs. placebo on synchronisation indices during tapping to computer, bidirectional and unidirectional tapping. *Indicates a significant effect at p < 0.05, ** at p < 0.001. Error bars represent standard error of the mean.

**Figure 3:**
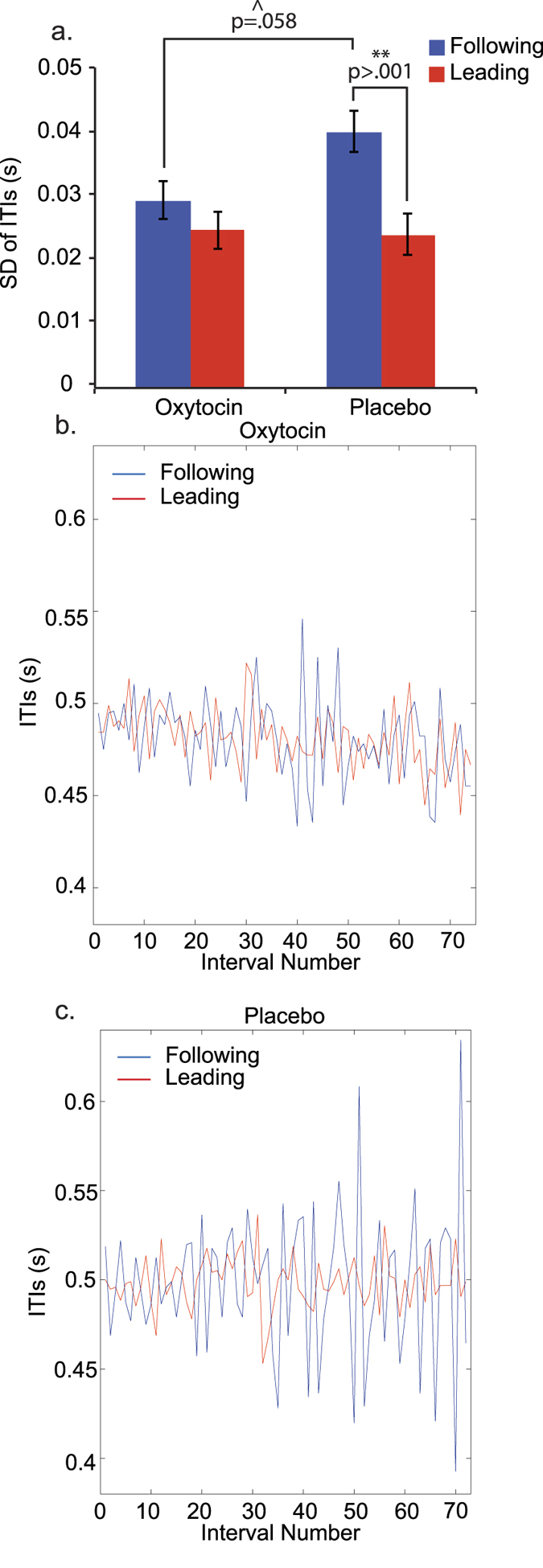
Effect of oxytocin vs. placebo on tapping variability. (**a**) Standard deviation (SD) of inter-tap intervals (ITI) for leading (tap to self) and following (tap to unresponsive other) during unidirectional tapping. **Indicates a significant contrast p < 0.001. ^Indicates a marginally significant effect. Error bars represent standard error of the mean. (**b**) Example of tapping dyad given oxytocin during unidirectional tapping, showing reduced following variability. (**c**) Example of dyad given placebo during unidirectional tapping, showing greater following variability.

**Figure 4:**
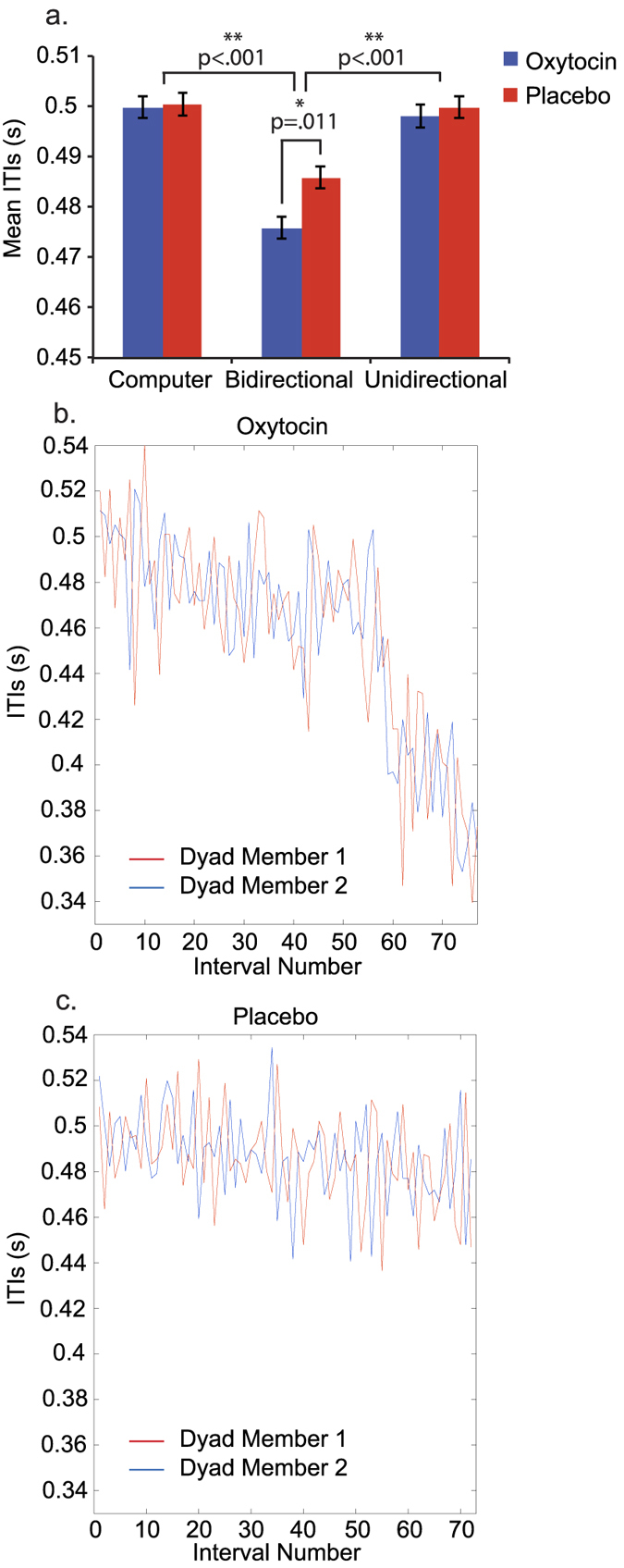
Effect of oxytocin vs. placebo on tapping speed. (**a**) Mean inter-tap interval (ITI), during tapping to computer, bidirectional and unidirectional tapping. *Indicates a significant contrast at p < 0.05, ** at p < 0.001. Error bars represent standard error of the mean. (**b**) Example of tapping dyad given oxytocin during bidirectional tapping, showing gradual increase in tapping speed. (**c**) Example of tapping dyad given placebo during bidirectional tapping, showing little change in tapping speed over time.

**Figure 5:**
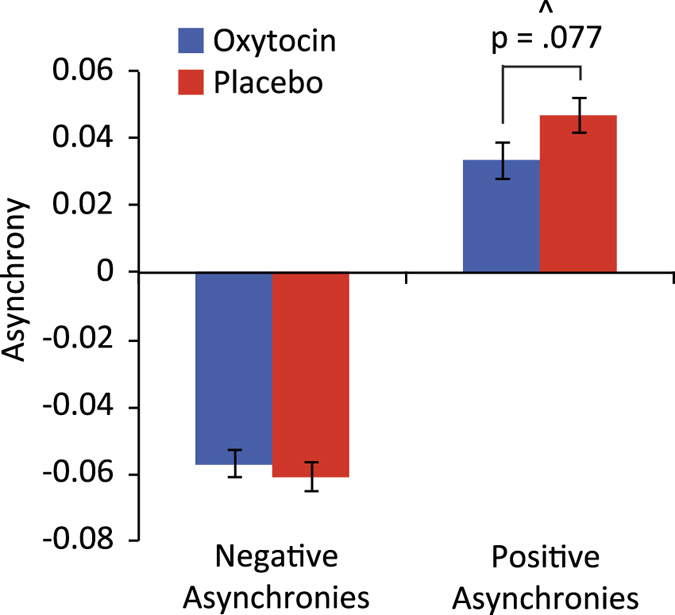
Effect of oxytocin vs. placebo on negative and positive asynchronies. ^Indicates a marginally significant effect. Error bars represent standard error of the mean.

During the second part of the experiment, dyad members were placed in two separate rooms, eliminating visual and external auditory feedback. A different experimenter to the one that had provided the nasal spray was responsible for administering the tapping tasks. Tapping was recorded for the following three conditions, which varied in degree of social interaction (Fig. 1):

- Computer metronome/non-social tapping. Both only hear completely regular (with no variability) computer-generated beats at 120 bpm.
- Bidirectional coupling/tapping to responsive other responsive other. Both participants hear beats generated by the other participant, but not their own tapping.
- Unidirectional coupling/tapping to unresponsive other. Both participants hear only tapping of either member one or member two, creating a leader-follower relationship. In half of the trials, member one taps to self while member two taps to member one. In the other half, this relationship is inversed.

Approximately 45 min after nasal spray administration, participants began the tapping experiment. Both dyad members were asked to tap with the index finger of their dominant hand for 17 bars (i.e. 68 beats) by pressing the keys corresponding to notes C3 and E3, respectively, following the initial 8-beat metronome. This comprised one trial of the experiment. Two different notes were chosen to allow participants to identify the sounds as coming from oneself or the other. Participants were told to start tapping as soon as they heard the metronome. Before each trial, the experimenter notified participants what they would be tapping to: 1) computer; 2) self, or 3) other. Participants were not told whether the other participant would be tapping to them or not (i.e. whether the coupling would be bidirectional or unidirectional). This was to ensure that their responses were unaffected by their beliefs about the directionality of the interaction and were instead fully a product of the interaction itself. The following instructions were given: “when hearing computer or other, continue tapping to the beat established by the initial metronome, while at the same time synchronising with the computer or the other member, respectively; when hearing self, continue tapping to the beat established by the initial metronome”. Each condition was carried out four times in semi-random order. The tapping part of the experiment lasted approximately 20 min. The study was double-blinded: neither participants nor the experimenter leading the tapping task knew whether dyads had been given OT or placebo.

### Pre-processing

Data were pre-processed for the dyadic analyses of interpersonal interaction (i.e. synchronisation indices, windowed cross-correlations, asynchronies), by ensuring that taps were aligned between pairs^54^. Thus, if one pair member skipped a beat, the tap of the other was removed, to ensure the shifts in the lagged cross-correlations were not artefactual. Similarly, if they drifted away from the given tempo (i.e. hear-self condition), we kept them aligned to each other’s closest beats in time, and removed a beat as soon as the other advanced in period. The maximum difference allowed in aligned taps between the two members was half of the average period (i.e. since the tempo was 120 bpm they could not be misaligned by more than 250 ms).

### Analysis

Five types of measures were computed from the data; synchronisation indices (SI), standard deviation (SD) of inter-tap intervals (ITIs), mean ITIs, asynchronies and windowed cross-correlations.

One way of measuring the strength of synchronization is to calculate synchronisation indices (SIs), which are quantified statistically using the distribution of phase difference between two signals^55^. SIs are based on variance of relative phase^56^, calculated with the formula in equation (1):


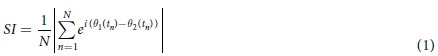


where *N* is the number of taps in each trial. ***θ**1* and ***θ**2* are the respective phases of each member in the dyad (or the participant and the computer in the computer condition). t_n_ corresponds to each discrete tapping instance. In other words, ***θ***1(t_n_) is the phase for tap 1, and ***θ***2(t_n_) is the estimated phase for tap 2, etc. The index is a unitless number, which ranges from 0 to 1, representing the absence of synchronisation and perfect synchrony, respectively.

SIs were calculated for dyads of participants in the unidirectional and bidirectional conditions, i.e. an SI value represents the synchronisation between the two members of a given dyad. For the bidirectional condition, dyad SI represented the synchronisation between the two dyad members averaged across trials. For unidirectional tapping, dyad SI was obtained by averaging both across trials and across leading and following conditions (i.e. the measure represented synchronisation regardless of whether it was member 1 or member 2 who was following or leading). SIs were calculated individually for the computer condition, between the individual tapper and the regular 120 bpm computer metronome. Here, individual SIs were averaged first across trials and then across the two dyad members to obtain the dyad synchronisation.

ITIs were computed for each trial and averaged for each participant (OT N = 50, Placebo N = 48). SD of ITIs was used as a measure of individuals’ tapping variability. For the unidirectional condition, data were split into ‘leading’ (tap to self) and ‘following’ (tap to unresponsive other). This ‘interacter-role’ was a within-subjects variable, since both dyad members were given the opportunity to act as both leader and follower. This allowed us to test the extent to which, when controlling for self-paced tapping variability, OT improves following variability. The effects of oxytocin and condition on mean ITIs indicated tapping speed. Here, the analysis unit was individuals’ mean ITI (OT N = 50, Placebo N = 48). For unidirectional mean ITIs, the values were averaged across leading and following.

Negative and positive asynchronies (negative and positive error in seconds from tapping target) were computed to investigate the extent to which tappers anticipated or reacted to tapping targets, respectively. We focused on the unidirectional condition, since only this condition had shown differences in synchronisation and variability. Furthermore, we did not analyse asynchronies in the bidirectional condition where tappers were constantly switching between leading and following (as could be seen in the windowed cross correlations). In other words, it would be near impossible to determine whether it was member 1 or member 2 who was leading or following and thus impossible to determine whether the asynchrony was positive or negative for the given tapper. In the unidirectional condition, there was a more stable pattern of leading and following, and thus asynchronies could be calculated for this condition. Positive and negative asynchronies were calculated independently, so the measures are representative even though participants shifted between premature/anticipatory and reactive modes of tapping. For each trial, the average negative asynchrony was calculated by summing the negative asynchronies and dividing by the number of negative asynchronies. The same was done for positive asynchronies. Finally, the data were averaged across trials, representing the average negative and positive asynchrony for each participant (OT N = 50, Placebo N = 48) when acting as follower (the leader would have the inverse of the follower’s asynchrony). Also the number of taps associated with positive and negative tapping errors were compared between the two groups, and variability (SD) of absolute asynchronies was computed, to investigate the stability of tapping performance. We performed correlations with the asynchrony data and SIs, and compared correlation coefficients between the two groups using Fisher’s r-to-z transformations.

In order to quantify how much each participant adapted to their partner based on their partner’s previous ITI, lag −1, 0 and +1 correlation coefficients were computed from a cross-correlation between the ITIs of the two members in each dyad^57^. Following the procedures reported in Konvalinka *et al*.^31^, we used a moving window size of 6 taps, a maximum lag of 3, and a window and lag increment of 1. The correlation coefficients for lag −1, 0, and +1 were computed between the participants’ ITIs across these short intervals of time and were averaged per condition for each pair. This analysis gave an indication of the directionality of the interaction – namely, whether the participants were not interacting with each other (i.e., uncorrelated), whether there was a clear leader–follower dynamic where one participant led the other towards their own tempo, or whether the adjustment of ITIs was mutual. For example, a positive lag −1 correlation alone would indicate a leader–follower dynamic such that one member is adjusting to the previous tap of the other. This means that if the ‘leader’ went faster on the previous movement tap, the ‘follower’ would speed up on the next one. Similarly, a positive lag +1 correlation would indicate the other member is adapting (and thus is the follower). A positive lag 0 correlation would mean that the participants are correlated in real time, indicating that when one speeds up, the other speeds up as well; finally, a negative lag 0 correlation would mean that the two participants were anti-correlated, such that when one speeds up, the other slows down^54^,^58^. The coefficients were compared across groups using 2 (OT vs Placebo) × 4 (hearing self; member 1 acting as leader, member 2 acting as follower; member 1 acting as follower, member 2 acting as leader; bidirectional coupling) multivariate analysis of variance (MANOVA) and lag −1, 0, and +1 correlation coefficients as the dependent variables.

Some participants failed to complete one, both or all the questionnaires. Where participants had incorrectly left blank responses or ticked more than one response, we replaced the data with their average score.

### Statistical Analysis

Statistical analyses were performed in R (except for windowed cross-correlations, which were analysed in MATLAB). To examine the effects of oxytocin, conditions and personality on our various measures, separate linear mixed models were used (LMM). For synchronisation indices (SI) pairs were entered as a random factor, since data points represented average dyad synchronisation (OT = 25, Placebo = 24). The dyadic nature of these data meant that we could not include SQ and TAS scores in the analysis for SI, since the scores represented individual’s personality and not dyad’s. The following fixed factors were specified for SI: group, condition, group × condition.

For variability (SD ITIs), we first investigated the computer and bidirectional conditions. Individual subjects were specified as a random factor, and group, condition, group × condition, group × SQ and group × TAS were set as fixed factors. For the unidirectional condition, data were split into ‘leading’ (tap to self) and ‘following’ (tap to unresponsive other). This ‘interacter-role’ was a within-subjects variable, since both dyad members were given the opportunity to act as both leader and follower. Individual subjects were the random factor, and the following were specified as fixed factors: group, role, group × role, group × SQ and group × TAS.

For tapping speed, we specified the LMM with individual subjects as the random factor and the following as fixed factors: group, condition, group × condition, group × SQ and group × TAS. Finally, for asynchronies, we investigated positive and negative asynchronies separately, with subjects as the random factor and group, group × SQ and group × TAS were the fixed factors.

We first tested the full models, and depending on the significance of the results, reduced the models to include only factors that had a significant effect on the outcome variables. The full models were compared to the reduced models with an ANOVA and with Akaike’s Corrected Information Criterion (ACI). If the ANOVA showed no significant improvement of the more complex model, and the ACI was lower for the reduced model, we reported the F-ratios and performed subsequent post-hoc Bonferroni corrected comparisons on the reduced model. In cases where the models were reduced to no longer include the SQ and TAS scores, the model was rerun with the full number of subjects N = 98 (i.e. for tapping speed and both asynchrony measures).

## Additional Information

**How to cite this article**: Gebauer, L. *et al*. Oxytocin improves synchronisation in leader-follower interaction. *Sci. Rep.* **6**, 38416; doi: 10.1038/srep38416 (2016).

**Publisher's note:** Springer Nature remains neutral with regard to jurisdictional claims in published maps and institutional affiliations.

## Figures and Tables

**Table 1:** Effect of oxytocin on the dyads’ synchronisation indices.

Synchronisation Indices		
	F(df)	p-value
Main effect of condition	43.65 (2,94)	<0.001**
Main effect of group	4.30 (1,47)	0.044*
Condition-by-group interaction	4.14 (2,94)	0.019*

Note: *Significant at 0.05, **Significant at 0.001.

**Table 2:** Participants’ descriptive statistics and characteristics.

	Oxytocin N = 50	Placebo N = 48	*t*(96)	*p*-value
Age	22.9	24.3	−2.157	0.034
(SD/range)	(3.15/18–33)	(3.24/19–33)		
Handedness	45 R/5 L	41 R/7 L		ns*
MET^1^ rhythmic	36.34	36.00	0.324	*ns*
(SD/range)	(4.96/25–47)	(5.42/25–46)		

Note: *Chi Square test. *ns* = not significant at α = 0.05. ^1^The rhythmic subscale of the Musical Ear Test.
